# Prevalence of low high-density lipoprotein among young adults receiving antiretroviral therapy in Zambia: An opportunity to consider non-communicable diseases in resource-limited settings

**DOI:** 10.1371/journal.pone.0247004

**Published:** 2021-02-16

**Authors:** Benson M. Hamooya, Patrick Musonda, Wilbroad Mutale, Sepiso K. Masenga, Hikabasa Halwiindi, Katongo H. Mutengo, Kaseya O. R. Chiyeñu, Gershom Chongwe, John R. Koethe, Loren Lipworth, Douglas C. Heimburger

**Affiliations:** 1 University of Zambia School of Public Health, Lusaka, Zambia; 2 Mulungushi University School of Medicine and Health Sciences, Livingstone, Zambia; 3 Vanderbilt Institute for Global Health, Nashville, TN, United States of America; 4 Department of Biomedical Sciences, University of Zambia School of Health Sciences, Lusaka, Zambia; 5 Vanderbilt University Medical Center, Nashville, TN, United States of America; 6 Livingstone Central Hospital, Livingstone, Zambia; 7 Ministry of Health, Lusaka, Zambia; 8 Vanderbilt University School of Medicine, Nashville, TN, United States of America; 9 University of Zambia School of Medicine, Lusaka, Zambia; Shanghai Jiao Tong University, CHINA

## Abstract

**Background:**

With the introduction of effective antiretroviral therapy (ART), people living with HIV (PLWH) are surviving longer and are at risk for developing metabolic abnormalities that contribute to cardiovascular disease (CVD). In Sub-Saharan Africa (SSA), there is a paucity of epidemiological data on lipid profiles among young adults receiving ART. This study aimed to estimate the prevalence of low high-density lipoprotein cholesterol (HDL-c), a cardioprotective lipid class, and whether it differed by age among adults on ART in Livingstone, Zambia.

**Methods:**

From April to December 2019, we conducted a cross-sectional study of 597 PLWH [n = 58 aged 18–24 years (young adults); n = 539 aged ≥25 years (adults)] on ART for ≥6 months. Data collected included demographic and lifestyle information, anthropometrics, viral load (VL), CD4 count, blood pressure, lipid profiles and fasting/random blood glucose. Clinical measures were defined as: low HDL-c [<1.0 mmol/L for men, <1.3 for women], increased waist circumference (WC) [≥94 cm for men, ≥80 cm for women], high triglycerides (TG) [≥1.7 mmol/l], and virological failure (VF) [VL ≥1000 copies/μl]. We used logistic regression to examine the association between age and low HDL-c after adjusting for multiple variables.

**Results:**

Among the young adults, 60% (35/58) were women, median (25^th^, 75^th^ percentile) age 21 years (18, 23), and median time on ART 116 months (60, 144). Among adults, 63% (342/539) were women, median age 46 years (40, 53) and median time on ART 108 months (60, 144). Young adults had a lower CD4 count compared to adults (median, 492 vs. 568 cells/μL, p = 0.010) and higher prevalence of VF (29% vs. 17%, p = 0.016). In young adults, prevalence of low HDL-c was significantly higher than in adults (63 vs. 38%, p<0.001). A high proportion of young adults (75%) and adults (58%) with low HDL-c were on dolutegravir (DTG)-based ART regimens. After adjusting for sex, duration on ART, WC, body mass index, ART regimen, VF, CD4 count, low density lipoprotein cholesterol, blood pressure and smoking, young adults were significantly more likely than adults to have low HDL-c (odds ratio 2.93; 95% confidence interval 1.46–5.86).

**Conclusion:**

Low HDL-c is highly prevalent among young adult with HIV in SSA independent of other risk factors for metabolic derangements. Lipid abnormalities among young PLWH may contribute to the early development of cardiovascular diseases in this population. This highlights the need to consider low HDL-c in the quest to reduce CVD risk among young adults on ART in SSA.

## Introduction

With the introduction of effective antiretroviral therapy (ART), people living with HIV (PLWH) survive longer [1] and are at risk for developing metabolic abnormalities that contribute to cardiovascular disease (CVD) [2–4]. A systematic review and meta-analysis indicated that the risk of CVD in PLWH is double that of HIV-negative individuals [5]. Furthermore, it was shown that HIV-associated CVD accounts for 2.6 million disability-adjusted life-years (DALYs) annually, with the greatest effect in Sub-Saharan Africa (SSA). Atherosclerosis is a major contributor to cardiovascular morbidity and mortality [6]. Previous longitudinal studies have shown that a higher concentration of high-density lipoprotein cholesterol (HDL-c) is protective against CVD [7, 8], which can result from atherosclerosis [9]. Additionally, in the Framingham Heart Study, an increase in HDL-c of 0.26 mmol/L was correlated with a significant decrease in CVD mortality [10].

In low- and middle-income countries (LMICs), the potential burden of dyslipidemia among patients receiving ART is of public health concern. In SSA, varying results on prevalence of low HDL-c have been reported among ART patients, ranging from 15.9 to 83.3% [11–19]. However, these studies did not differentiate their estimates based on participant age.

Among young adults living with HIV in Europe, the prevalence of multi-morbidity (defined as having 3 or more of the following: CVD, hypertension, dyslipidemia, diabetes or chronic kidney disease) was 6.3% in one study [20]. In LMICs there is limited information on specific cardiovascular risk factors among young adults living with HIV. This poses a challenge in instituting targeted screening given the constraints in resources. The Bogalusa Study (United States), among children and young adults in the general population, revealed that HDL-c was associated with lesions in the aorta and coronary arteries [21]. In resource-constrained areas there is a need to find cheaper and specific ways of identifying individuals who would be at an increased risk of developing CVD, such as HDL-c.

The interaction between HIV infection, ART, and serum lipoproteins is not fully understood. Low HDL-c has been associated with high viral load [22] and ART [17, 23], but almost all reports are from adults, and they cannot necessarily be extrapolated to young adults. During the first 24 weeks of ART, a randomized controlled trial in South Africa observed a sharp increase in all lipid levels [24], indicating that lipid abnormalities may develop early on ART. Despite these findings, in SSA, monitoring for metabolic abnormalities is rarely done, and there is a need for information on the burden and risk factors associated with low HDL-c among young adults and adults receiving ART. This information could contribute to age-specific management and prevention of most common cardiometabolic risk factors in individuals on ART. Additionally, HDL-c has antiatherogenic effects [25], and estimating the prevalence of reduced HDL-c can help determine population risk for CVD and/or atherosclerosis. Therefore, this study aimed at estimating the prevalence of low HDL-c, a cardioprotective lipid class, whether it differed between young adults and adults, and assessing the factors associated with low HDL-c among individuals on ART in Livingstone, Zambia.

## Material and methods

### Study design and setting

We conducted a cross-sectional study among HIV-positive adults who were attending routine care and treatment at Livingstone Central Hospital Medical Clinic (LCHMC). The hospital is based in Livingstone city, a tourist destination in Zambia, which has an estimated population of 140,000. Livingstone Central Hospital is the largest referral hospital in the Southern Province of Zambia. All advanced ART care and treatment cases in the province are referred to LCHMC. Patients are given different clinic visit schedules (every 2 weeks- newly diagnosed, 3 months- unstable, and 6 months- stable on ART) for immuno-virologic and clinical evaluations and pharmacy refills. Currently, the facility has approximately 3,875 adult patients ≥18 years of age on ART.

### Study participants

The study was conducted between April and December 2019. We recruited 597 participants attending routine HIV treatment appointments. Eligible participants were individuals aged ≥18 years who had been receiving ART for 6 months or more. The threshold of ≥6 months was chosen because the effect of ART on serum lipid profile would likely have been established by then [26]. Young adults were defined as those aged between 18 and 24 years, and adults were ≥25 years old. Pregnant women, because of metabolic changes accompanying pregnancy such as increased LDL-c which could indirectly affect the findings, and patients with known active opportunistic infection or neoplasm were excluded from the study.

### Data collection

Data on demographic (age, sex, educational background, marital status, living conditions), clinical (ART regimen, blood pressure, height, weight, year diagnosed HIV-positive, perinatal HIV infection, adherence to ART), behavioral/lifestyle (alcohol consumption, smoking status and physical exercise) and laboratory (HIV type, lipid profile, CD4 count, glucose fasting and non-fasting, hepatitis, viral load) characteristics were collected directly from participants and from the medical record using a structured questionnaire (modified from the WHO STEPS survey questions [27]) and data collection form, respectively. The questionnaire was developed based on previously used templates and already existing questionnaires which were modified to suit the current study. To test understanding and completion of the current questionnaire, a pilot study was conducted among 20 individuals who met the inclusion criteria. The data collectors underwent three days of training to ensure reliability and accuracy of the information collected.

### ART-based regimens

- Non-nucleoside reverse transcriptase inhibitor (NNRTI) regimens contained efavirenz (EFV) or nevirapine (NVP) with one of the following nucleoside reverse transcriptase inhibitors (NRTIs):
  ○ Abacavir and lamivudine/emtricitabine (ABC/XTC) or tenofovir disoproxil fumarate and lamivudine/emtricitabine (TDF/XTC)
- Protease inhibitor (PI) regimens had either lopinavir/ritonavir (LPV/r) or atazanavir/ritonavir (ATZ/r) with one of the following NRTI combinations:
  ○ ABC/XTC or zidovudine/XTC (AZT/XTC) or TDF/XTC
- An Integrase strand transfer inhibitor (INSTI) regimen had dolutegravir (DTG) with TDF/lamivudine (TDF/3TC)

### Blood samples and measurements

Samples were collected from the study participants in fasting and non-fasting states. For the tests requiring fasting blood (lipids and fasting blood glucose [FBG]), participants were given another appointment to come fasting. Blood samples in vacutainer tubes were clearly labeled and transported immediately to the laboratory for analysis in a cooler box. The samples were assayed within 3 hours of collection. The samples for total cholesterol (TC) (mmol/l), HDL-c (mmol/l), LDL-c (mmol/l) and triglycerides (mmol/l) were collected in green topped containers (lithium heparin). In the laboratory, the samples were centrifuged for 10 minutes at 10,000 relative centrifugal force. The centrifuged samples were analyzed using a HumaStar 600 machine. Samples for FBG (mmol/l) were collected by finger pricking and analyzed using an Accu-check point of care machine. CD4 count (cells/μl) samples were collected in ethylenediaminetetraacetic acid (EDTA) containers and assayed using a Becton Dickson flow cytometer. Viral load (copies/mL) samples were collected in EDTA containers and analyzed using an Ampliprep/Taqman 96 PCR analyzer.

Blood pressure (using a digital machine- Omron-HEM-7120, USA) was determined by calculating an average of three readings obtained after resting participants for 5 minutes and taking readings one minute apart. The height (cm), weight (kg) and waist circumference were measured using a height measurement chart, digital scale and tape measure, respectively.

### Operational definitions

The following were defined according to harmonized criteria for abnormal levels [28]:

○ Waist circumference: ethnicity specific [Sub-Saharan African; ≥94 cm (male), ≥ 80 cm (female)]
○ TG ≥ 1.7 mmol/L, or specific treatment for this lipid abnormality
○ HDL-c < 1.0 mmol/L in males, < 1.3 mmol/L in females
○ FBG: ≥ 5.6 mmol/L, or previously diagnosed diabetes mellitus
○ Systolic BP ≥ 130 or diastolic BP ≥ 85 mmHg, or treatment of previously diagnosed hypertension

Abnormal TC (≥ 5.1mmol/l) and LDL-c (≥ 3.4mmol/l) were defined according to 2018 American College of Cardiology/American Heart Association Task Force on Clinical Practice Guidelines [29]. Virologic failure was defined as plasma HIV-1 RNA viral load (VL) of ≥1000 copies/ml [30].

Body mass index (BMI) was classified as underweight (<18.5 kg/m^2^), normal (18.5–24.9 kg/m^2^), overweight (25.0–29.9 kg/m^2^), or obese (≥30 kg/m^2^) [31]

A participant was considered to be physically active if:

○ Their typical week’s work involved carrying or lifting heavy loads, digging, crushing stones or construction work for at least 10 minutes continuously.
○ They participated in moderate or vigorous-intensity sports, fitness or recreational (leisure) activities in a typical week such as running, football, cycling, swimming, or volleyball for at least 10 minutes continuously

### Statistical analysis

All analyses were performed using STATA software, version 15.0/IC (Stata Corporation, College Station, TX, USA). Categorical data were summarized using frequencies and proportions. Continuous variables (e.g., age, time on ART and BMI) were summarized using medians and quantiles. Q-Q plots were used to determine the normality of the data. The Pearson chi-square test was used to test for statistically significant associations between two categorical variables *(Table 1)*. The non-parametric Kruskal Wallis test was employed to detect differences between HDL-c levels among three ART-based regimens (NNRTIs, PIs and INSTI) *(Fig 2)*. Logistic regression was used to estimate the odds ratios and 95% confidence intervals for the association between low HDL-c and age group alone (unadjusted model) and adjusting for sociodemographic, immuno-virologic, clinical and laboratory factors *(Table 2)*. Variables in the multivariable model were selected based on the findings of previous studies and those which were statistically significant in univariable analysis. No interactions were included in the model. Statistical significance was defined as p < 0.05.

**Table 1:** Sociodemographic, behavioral, physical and clinical factors sorted according to low HDL-c status.

Characteristic	*N*	Low HDL-c		p-value
		Yes, n = 238 (40%)	No, n = 356 (60%)	
***Sociodemographic*, *behavioral and physical***				
**Age category** *(years)*, *%*	597			**<0.001**
18–24 (young adult)		63	37	
≥25 (adult)		38	62	
**Sex**, *Female vs*. *male %*	597	46 vs. 30	54 vs. 70	**<0.001**
**Current smokers**, *yes vs*. *no (%)*	596	30 vs. 41	70 vs. 59	0.333
**Currently married**, *yes vs*. *no (%)*		35 vs. 46	65 vs. 54	**0.005**
**Education level** *(%)*	597			
No formal		40	60	0.142
Primary		34	66	
Secondary		44	56	
Tertiary		37	63	
**Work status** *(%)*	596			**<0.001**
Formal employment		38	62	
Self-employed		38	62	
Student		79	21	
Retired		22	78	
Unemployed		45	55	
**Living alone**, *yes vs*. *no (%)*	596	35 vs. 40	65 vs. 60	0.587
**Income above K1,500**, *yes vs*. *no (%)*	595	36 vs. 43	64 vs. 57	0.083
**Physical exercise**, *yes vs*. *no (%)*	592	43 vs. 39	61 vs. 57	0.377
**Clinical characteristic**				
**BMI category** *(kg/m*^*2*^*) (%)*				0.110
Underweight		36	64	
Normal weight		37	63	
Overweight		47	53	
Obese		49	51	
**CD4 absolute count** *(cells/μl)*, *%*	565			0.375
< 500		37	63	
≥ 500		41	59	
**Viral load (copies/μl)**, *%*	592			**0.021**
< 1000		38	62	
≥ 1000		50	50	
**Duration on Current ART**, *months %*	595			**<0.001**
≤ 12		53	47	
13–24		40	60	
≥ 25		34	66	
**ART regimen, %**	597			**<0.001**
NNRTI (EFV&NVP)		33	67	
PI (LPV/r&ATZ/r)		44	56	
INSTI (DTG)		59	41	
**Raised BP** *(mmHg)*, *yes vs*. *no (%)*	597	41 vs. 40	59 vs. 60	0.833
**Raised FBG** *(mmol/l)*, *yes vs*. *no (%)*	447	47 vs. 37	53 vs. 63	0.106
**Raised WC** *(cm)*, *yes vs*. *no (%)*	597	48 vs. 35	52 vs. 65	**0.001**
**Raised TG** *(mmol/l)*, *yes vs*. *no (%)*	594	41 vs. 40	59 vs. 60	0.770
**Raised LDL-c** *(mmol/l)*, *yes vs*. *no (%)*	568	26 vs. 41	74 vs. 59	**0.031**

N number of no-missing values, HDL-c high-density lipoprotein cholesterol, BMI body mass index, CD4 cluster of differentiation 4, *kg/m*^*2*^ kilogram per meter squared, ART antiretroviral therapy, NNRTI non-nucleoside/nucleotide reverse transcriptase inhibitor (EFV = efavirenz and NVP = Nevirapine), PI Protease inhibitor (LPV/r = lopinavir/ritonavir and ATV/r = atazanavir/ritonavir), INSTI integrase strand transfer inhibitor (DTG = dolutegravir), BP blood pressure, FBG fasting blood glucose, WC waist circumference, TG triglycerides, TC total cholesterol, LDL-c low density lipoprotein cholesterol.

**Table 2:** Association of low HDL-c with demographic, behavioral and clinical characteristics of study participants.

Variables	unadjusted analysis			adjusted analysis		
	OR	95%CI	p-value	OR	95%CI	p-value
**Age** *(18–24 vs*. ≥ *25y)*	2.84	1.61–5.01	**<0.001**	2.93	1.46–5.86	**0.002**
**Sex**, *Female vs*. *male*	1.96	1.38–2.79	**<0.001**	2.54	1.55–4.15	**<0.001**
**Current smokers**, *yes*	0.64	0.26–1.58	0.337	1.36	0.46–4.00	0.572
**Physical exercise**, *yes*	1.19	0.80–1.77	0.377			
**BMI category** *(kg/m*^*2*^*)*						
Underweight	1					
Normal weight	1.05	0.64–1.71	0.840	0.80	0.45–1.41	0.444
Overweight	1.55	0.88–2.73	0.128	0.88	0.41–1.86	0.737
Obese	1.72	0.89–3.33	0.104	0.89	0.35–2.22	0.806
**CD4 absolute count** *(cells/μl)*						
< 500	1					
≥ 500	1.17	0.83–1.64	0.375	1.09	0.74–1.62	0.652
**Viral load** *(copies/μl)*						
< 1000	1					
≥ 1000	1.64	1.07–2.50	**0.022**	1.70	1.02–2.82	**0.040**
**Duration on Current ART**, *months*						
≤ 12	1					
13–24	0.61	0.31–1.22	0.162	1.34	0.51–3.55	0.554
≥ 25	0.47	0.33–0.68	**<0.001**	1.37	0.60–3.11	0.449
**ART regimen**						
NNRTI (EFV&NVP)	1					
PI (LPV/r&ATZ/r)	1.56	0.95–2.58	0.080	1.72	0.82–3.61	0.150
INSTI (DTG)	2.93	1.93–4.45	**<0.001**	6.77	2.66–17.25	**<0.001**
**Raised BP**, *yes*	1.04	0.74–1.45	0.833	1.08	0.71–1.64	0.711
**Raised FBG**, *yes*	1.50	0.92–2.44	0.107			
**Raised WC**, *yes*	1.73	1.24–2.42	**0.001**	1.43	0.85–2.42	0.180
**Raised TG**, *yes*	1.07	0.69–1.64	0.770			
**Raised LDL**, *yes*	0.50	0.27–0.95	**0.033**	0.36	0.16–0.81	**0.014**

OR odds ratio, CI confidence interval, y years, BMI body mass index, CD4 cluster of differentiation 4, *kg/m*^*2*^ kilogram per meter squared, ART antiretroviral therapy, BP blood pressure, FBG fasting blood glucose, WC waist circumference, TG triglycerides, TC total cholesterol, LDL-c low density lipoprotein cholesterol, NNRTI non-nucleoside reverse transcriptase inhibitor, EFV efavirenz, NVP nevirapine, PI protease inhibitor, LPV/r lopinavir/ritonavir, ATZ/r atazanavir/ritonavir, INSTI integrase strand transfer inhibitor, DTG dolutegravir.

### Ethical considerations

Ethical approval for the study was obtained from University of Zambia Biomedical Research Ethics Committee (UNZABREC- REF. No. 003-12-18). To all the participants the purpose of the study was explained in the language they understood, and they gave written informed consent.

## Results

### Descriptive characteristics

The study comprised 597 participants of whom 58/597 (10%) were aged 18 to 24 years (young adults). Median (25^th^, 75^th^ percentile) ages in young adults and adults (≥25 years) were 21 years (18, 23) and 46 years (40, 53), respectively. Young adults had total median time on ART of 116 months (60, 144), and a majority of them had been on their current ART regimen for ≥25 months 30/58 (52%). Among adults, the total median time on ART was 108 months (25^th^, 75^th^ percentile 60, 144), and 343/537 64% had been on their current ART regimen for ≥25 months. In both groups, the majority of participants were women [young adults-342/539 (60%) and adults-35/58 (63%)]. Overall median (25^th^, 75^th^ percentile) for BMI was 22.4 kg/m^2^ (19.6, 25.6), significantly higher in adults (22.7 vs. 20.1 kg/m^2^; p<0.001). The majority of participants were on NNRTI- based regimens 400/597 (67%); 75/597 (13%) were in a PI-based regimen and 122/597 (20%) were on the INSTI regimen.

### Relationships between the outcome (low HDL-c) and explanatory (sociodemographic and clinical) variables

Table 1 shows the relationships between low HDL-c and sociodemographic, behavioral, physical and clinical variables. The prevalence of low HDL-c was significantly higher in participants aged 18–24 years (63% vs. 38%, p<0.001). A higher proportion of females (46%) vs. males (30%), p<0.001 and those not currently married (46% vs. married 35%, p = 0.005) had low HDL-c. Students, when compared to other work statuses, had a high proportion of low HDL-c (79% vs. 38%, 22%, 45%, p<0.001). Individuals with VF (≥1000 copies/ml) had significantly higher proportion of low HDL-c (50% vs. 38%, p = 0.021). Participants on current ART regimen for ≤ 12 months (53%) had higher proportion of low HDL-c as compared with those on ART for 13–24 months- 40% and ≥ 25–34% (p<0.001). These results showed an evidence of a trend (p<0.001). Fifty nine percent (59%) of participants with low HDL-c were on the INSTI-based regimen, and this was significantly higher as compared with those on NNRTIs (33%) and PIs (44%), p<0.001. Low HDL-c was more prevalent among participants with raised waist circumference (48% vs. 35%, p = 0.001). The prevalence of low HDL-c was low among the participants with raised LDL-c (26% vs. 41%, p = 0.031).

### Relationship between HDL-c and ART regimen by study cohorts

**Fig 1:**
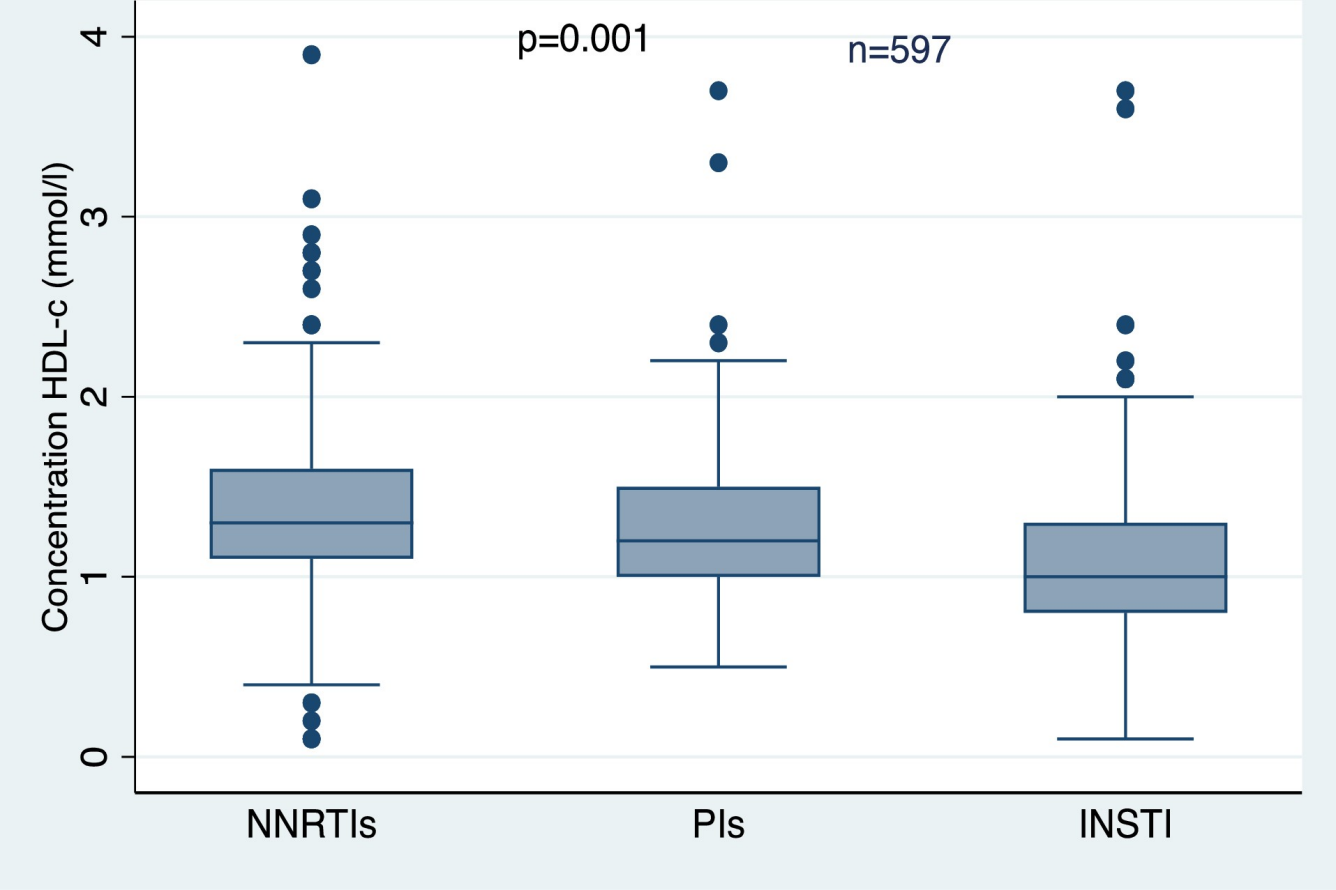
The medians of HDL-c in different ART regimens among all the participants.

**Fig 2:**
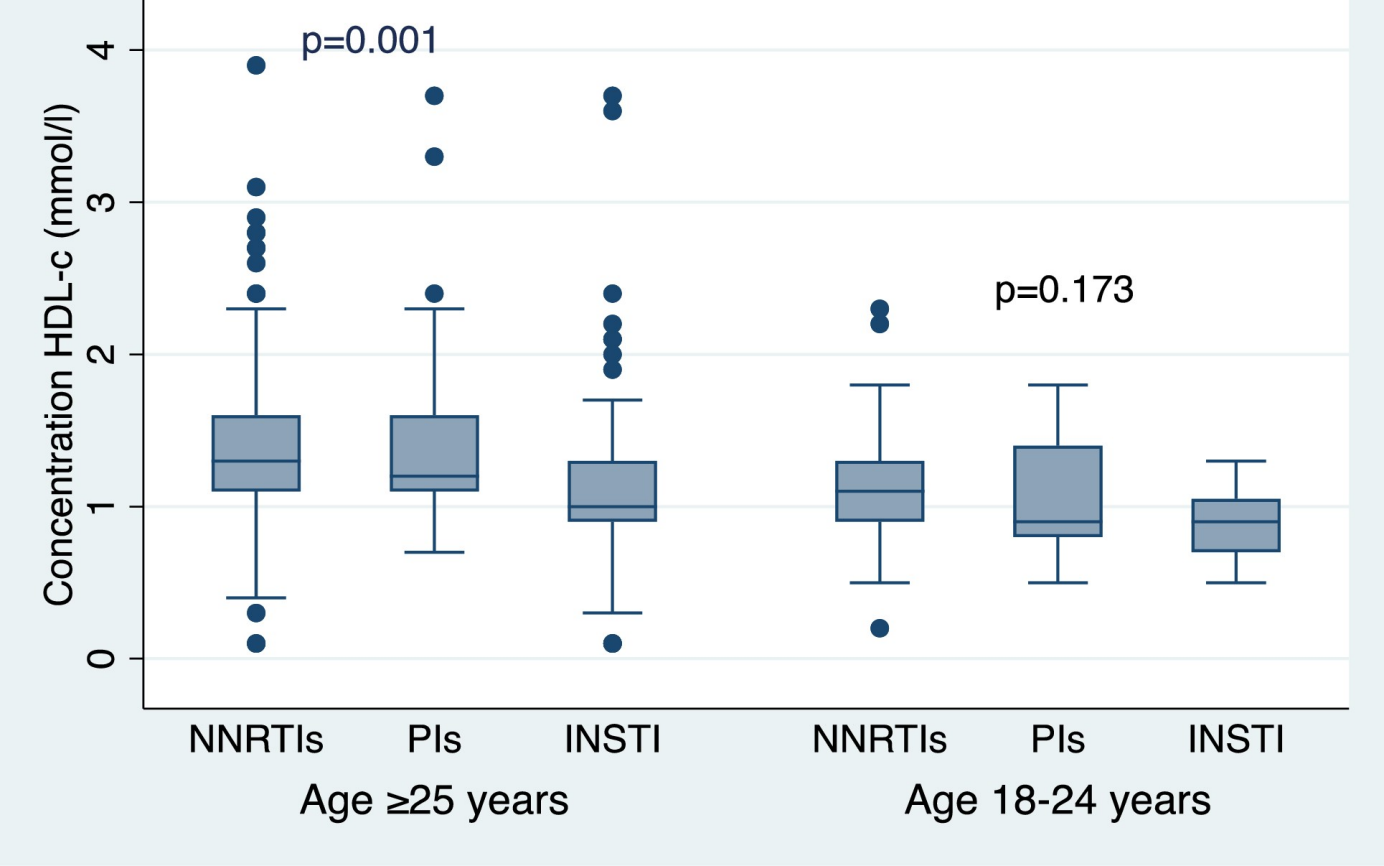
High-density lipoprotein cholesterol (HDL-c) stratified by ART regimen among the two study groups.

Among all study participants, HDL-c was significantly lower among those on the INSTI-based ART regimen (p = 0.001) *(see Fig 1)*. However, after sorting the data by age groups (18-24y and ≥25y), significantly lower HDL-c was observed only among those aged ≥25y on INSTI (p = 0.001). Young adults on PIs and INSTI-based regimen had lower HDL-c, although the findings were non-significant (p = 0.173) *(see Fig 2)*.

### Factors associated with Low HDL-c

Table 2 shows results of unadjusted and adjusted logistic regression models of factors associated with low HDL-c. In unadjusted analysis we observed that patients with raised waist circumference and on current ART for ≥ 25 months had 73% [1.73 (OR), (1.24–2.42) 95% CI, p = 0.001] increased and 53% [0.47 (OR), (0.33–0.68) 95% CI, p<0.001] reduced odds of having low HDL-c, respectively. However, these findings were not statistically significant when controlling for other covariates. In multivariable logistic regression models (adjusted for age group, sex, smoking status, BMI, CD4 count, viral load, duration on current regimen, ART regimen, BP, WC and LDL-c), young adults and females were 2.93 (95%CI 1.46–5.86; p = 0.002) and 2.54 (95%CI 1.55–4.15; p<0.001) times more likely to have low HDL-c compared with adults and males, respectively. Participants with VL ≥1000 copies/μl had 70% [1.70 (OR), (1.02–2.82) 95% CI, p = 0.040] increased odds of low HDL-c. Moreover, individuals with elevated LDL-c were approximately 64% [0.36 (OR), (0.16–0.81) 95% CI, p = 0.014] less likely to have low HDL-c compared with those with normal LDL-c values. People on the INSTI-based regimen had 6.77-fold higher odds of having low HDL-c in comparison with those on NNRTI-based regimens.

## Discussion

In this study among young adults and adults with HIV receiving ART in Livingstone, Zambia, we found that 63% of young adults had low HDL-c, compared to 38% of adults. We further established that age group, female sex, virologic failure, and ART regimen (INSTI) were significant predictors of low HDL-c among our study participants.

After controlling for multiple potential confounders, young adults had almost three-fold higher odds of low HDL-c when compared to adults. Our findings are similar to a study in rural Uganda, which found age <30 years was associated with increased odds of low HDL-c [32]. However, our study population of young adults and adults on long-term ART differed from the study in Uganda, which compared healthy adults with HIV-positive adults who were not on ART.

Longer duration of ART treatment was not associated with low HDL-c in multivariable analyses. However, there was an evidence of a decreasing trend in the prevalence of low HDL-c with increase in duration of one being on a particular ART-regimen. Earlier reports showed that long-term duration of ART is associated with dyslipidemia and CVD complications [33, 34]. Previously in Sub-Saharan Africa, longitudinal studies have shown that HDL-c levels increased with longer duration of ART [24, 35, 36]. For instance, in rural Uganda mean HDL-c was significantly higher after 24 months of ART as compared to baseline [35]. This may be due to a number of factors such as effect of an aging HIV-positive population combined with viral suppression and the influence of some of the antiretroviral drugs on lipid metabolism.

We observed a significant association of the INSTI (DTG)-based regimen with lower HDL-c. However, in phase IIb-IIIb clinical trials, DTG showed a safer lipid profile (HDL-c, LDL-c and TC) in combination with NRTIs at 48 weeks [37]. Similarly, an observational cohort study in Italy observed improvement in lipid profiles after 48 weeks of switching patients to a DTG-based (ABC/3TC/DTG) regimen [38]. The inconsistency in results could be attributed to differences in the races, as the previous studies were predominantly among whites. In the current study, all the participants were black Africans of which genetics and behavioral factors like diet could be implicated in the observed differences. Hence there is need to have race-specific data on different ART-regimens. Furthermore, the study by Bagella et al. [38] had a smaller sample size of 131, which might have impacted the findings.

Protease Inhibitor (PI)-based regimens also showed poor HDL-c outcomes among our study participants as compared with NNRTIs. In the United States, a study in women also revealed that NNRTIs (NVP & EFV) were associated with higher HDL-c [39]. Similar findings were observed by Bernal et al. [40], where NNRTIs were protective against low HDL-c levels. There is support from cross-sectional and longitudinal studies indicating that PIs negatively affect lipid profiles [41–45]. However, there is a paucity of comparisons of INSTI- and PI-based regimens in different geographical areas. All in all, the issue of DTG’s effects on lipid profiles in SSA requires further investigation.

We also found that participants who had VF had lower HDL-c. Previously it has been shown that HIV-1 lowers plasma HDL by damaging the cholesterol-dependent efflux transporter ATP-binding cassette protein A1 in macrophages, and this condition increases risk of atherogenesis [45–47]. This mechanism could help explain why VF was significantly associated with low HDL-c; high viral load may significantly lower the plasma level of HDL. Additionally, it is possible that low HDL-c was more prevalent in young adults because of higher prevalence of VF as compared with adults. Our findings are consistent with previous studies that found high viral load associated with high prevalence of low HDL-c [16, 22].

In previous studies in Tanzania [12] and the United states [48], low HDL-c was more prevalent among men. These findings are not consistent with our findings, in which women had increased odds of having lower values of HDL-c. Similar results were observed in Ethiopia [49], Côte d’Ivoire [16] and India [22]. The discrepancies could have arisen from differences in the criteria used to define abnormal HDL-c. For instance, in a study by Ombeni and Kamuhabwa, the National Cholesterol Education Program Adult Treatment Panel (NCEP ATP) III criteria were used, which stipulates that HDL-c levels of 1.04 mmol/l or less in both men and women should be considered low [50]. This might impact the results given that women naturally have higher concentrations of HDL-c than men due to estrogen, which has been shown to increase plasma levels of HDL-c [51]. Furthermore, estrogen protects from atherosclerosis through the reduction of lipid accumulation in macrophages [52].

In terms of relationship with HDL-c with other lipid profiles, we observed a significantly higher prevalence of low HDL-c among individuals with normal values of LDL-c. This was further confirmed in the multivariable analysis, where participants with raised LDL-c were significantly less likely to have low HDL-c, i.e., a positive relationship between HDL-c and LDL-c as found by Kotani et al. [53]. We also found that a higher proportion of participants with raised WC had abnormal HDL-c. In rural China, similar results were observed in which individuals with normal WC had significantly higher HDL-c compared with those with abnormal WC [54]. These findings highlight the potential benefit of reducing WC through exercise and/or diet in achieving desirable levels of HDL-c and preventing dyslipidemia.

In this cross-sectional study, we could not determine the temporal relationship between low HDL-c and the covariates. However, there are well established statistical methods to establish predictive index and potential causal effect such as deep learning machine [55–57] and Mendelian randomization [58, 59]. But we could not use them due to the aim of this current study, lack of independent datasets and genetic data in our setting. The sample size in young adults was minimal and this could have reduced the power. There was also no HIV-negative control group to compare the prevalence of low HDL-c. However, the study was able to highlight the burden and factors associated with low HDL-c among persons with HIV, who are disproportionately affected by cardiovascular risk factors as compared to individuals without HIV [60].

## Conclusions

Our findings indicate that low HDL-c is prevalent in adults living with HIV, especially among young adults. Low HDL-c among individuals taking ART was related to viral load, ART regimen, LDL-c, younger adult age, and sex. The use of dolutegravir (INSTI) was associated with lower values of HDL-c, and this could increase the risk for CVD. Our findings suggest the need for routine examination of lipoprotein profiles at ART initiation and throughout ART, especially HDL-c in young adults. This study was a cross-sectional study, hence we could not make causal inferences, so a cohort study would be appropriate to assess HDL-c levels as patients start ART and observe outcomes such as CVD among young HIV-infected people in our setting.
